# Evaluating Clinical Practice: An Audit of Ophthalmologic Findings in Patients With Head and Neck Diseases in Otorhinolaryngology

**DOI:** 10.7759/cureus.92636

**Published:** 2025-09-18

**Authors:** Saleh Khurshied, Hira G Shah, Sana A Chohan, Muhammad Shuaib Khan, Mehrun Nisa, Muhammad A Zahid

**Affiliations:** 1 Otolaryngology-Head and Neck Surgery, Pakistan Institute of Medical Sciences, Islamabad, PAK; 2 Ophthalmology, Alshifa Trust Eye Hospital, Rawalpindi, PAK; 3 Otolaryngology-Head and Neck Surgery, Watim Medical College and General Hospital, Islamabad, PAK; 4 Otolaryngology-Head and Neck Surgery, Rawalpindi Medical University, Rawalpindi, PAK; 5 Medicine and Surgery, Pakistan Institute of Medical Sciences, Islamabad, PAK; 6 Ophthalmology, Monash Health, Clayton, AUS

**Keywords:** clinical symptoms, common symptoms, eye signs, ophthalmology, otorhinolaryngology and neck surgery

## Abstract

Background and objectives

Due to the close anatomical relationship between the eye and the ear, nose, and throat region, orbital involvement and related ophthalmologic symptoms are frequently encountered in the field of otorhinolaryngology-head and neck surgery. Understanding this connection is essential for accurate diagnosis, predicting disease progression, and planning effective treatment. This retrospective review explores the occurrence and patterns of ocular manifestations arising from primary otorhinolaryngology and head and neck conditions.

Material and methods

We conducted a retrospective review of discharge records of patients managed at the Department of Otorhinolaryngology-Head and Neck Surgery, Pakistan Institute of Medical Sciences, Islamabad, between April 2020 and July 2025. The study included only hospitalized adult patients, more than 18 years of age, who were diagnosed with otorhinolaryngology-head and neck conditions and exhibited associated ophthalmologic signs and symptoms. Data collected included patient demographics, clinical presentations, and confirmed diagnoses. The observed trends were summarized and presented using tables and figures.

Results

The mean age of the study population was 47 ± 21.5 years. Of the 98 patients, 67 (68.37%) were male and 31 (31.63%) were female. The most frequently observed otorhinolaryngologic condition associated with ophthalmologic manifestations was chronic rhinosinusitis (CRS), found in 12 cases (12.24%). This was followed by orbital cellulitis in 11 cases (11.22%), chronic otitis media (COM) with complications in 10 cases (10.20%), and both trauma and acute otitis media (AOM)/mastoiditis in eight cases each (8.16%). The most common ophthalmologic manifestation was epiphora, reported in 44 instances (18.80%), followed by chemosis in 39 cases (16.66%).

Conclusions

Epiphora and chemosis are the most frequently observed ophthalmologic manifestations in patients with otorhinolaryngology-head and neck diseases. Among these conditions, chronic rhinosinusitis and orbital cellulitis are the most common ear, nose, and tongue (ENT) disorders presenting with ophthalmologic signs and symptoms.

## Introduction

Due to its close anatomical proximity to the ear, nose, and throat (ENT) region, involvement of the orbit and associated ophthalmological manifestations are fairly common in the practice of otorhinolaryngology. Orbital complications may arise from infections in the “dangerous area” of the face, direct extension of pathologies from the nose, paranasal sinuses, and nasopharynx into the orbit, involvement of the nerves supplying the orbit and adnexa, or deposition of retro-orbital fat as seen in conditions like exophthalmic goiter [1].

The anatomical relationship between the nose, paranasal sinuses, and the orbit is important not only for understanding symptoms and disease spread but also for planning effective management strategies [2]. Orbital involvement in sinonasal diseases can present with ophthalmoplegia, proptosis, or even blindness due to optic nerve damage. Such involvement often indicates an extensive and aggressive pathology, and many of these conditions, even when benign, can be challenging to treat [3].

In fact, orbital involvement is frequently the initial presentation in a large proportion of paranasal sinus tumors. Ophthalmological manifestations associated with primary ENT diseases are common and can lead to serious complications. The significance of thorough clinical examination in preventing complications, including permanent blindness, following orbital involvement from sinusitis has been recognized worldwide [4].

The orbit is often affected in diseases of the nose and paranasal sinuses, primarily because approximately two-thirds of the osseous orbital wall is formed by the sinus walls [5]. Additionally, valveless venous communications within the orbital walls may contribute to the spread of infections or pathology to this region [6].

This study aims to evaluate the ophthalmological manifestations associated with primary ENT disorders in patients admitted to the Otorhinolaryngology-Head and Neck Surgery inpatient ward. We seek to determine the most common ophthalmological signs and symptoms associated with these conditions. The rationale behind this study is to provide essential insights into the ophthalmologic manifestations of otorhinolaryngology-head and neck diseases. By identifying the prevalent signs, symptoms, and associated conditions, this research will help clarify the frequent ophthalmo-otorhinolaryngological associations encountered by clinicians. This understanding is vital, as these overlapping manifestations often create diagnostic challenges and impact patient care and management.

## Materials and methods

This audit included patients of all ages who were admitted to the Otorhinolaryngology-Head and Neck Surgery inpatient department at the Pakistan Institute of Medical Sciences (PIMS), Islamabad, Pakistan, with otorhinolaryngological diagnoses accompanied by ophthalmological manifestations. The study period spanned five years, from April 2020 to July 2025. PIMS is a tertiary care government hospital with 46 beds designated for ENT and ophthalmology patients. Patients with primary ophthalmological diseases presenting with otorhinolaryngological signs and symptoms, those not admitted to the Otorhinolaryngology-Head and Neck Surgery inpatient department, and those with incomplete or missing data were excluded from the study.

Data, including age, gender, presenting signs and symptoms, and formal diagnoses, were retrospectively extracted from patient records, such as history sheets, examination notes, and discharge summaries. Formal written consent was obtained from the departmental head to use departmental data for this research. To ensure patient confidentiality, all identifying information was anonymized during data collection, and no direct contact was made with patients or their families.

Statistical analysis was performed using IBM Corp. Released 2018. IBM SPSS Statistics for Windows, Version 27.0. Armonk, NY: IBM Corp. Variables were summarized using frequencies and percentages. Descriptive data were presented in tables and charts to facilitate visualization.

## Results

**Figure 1:**
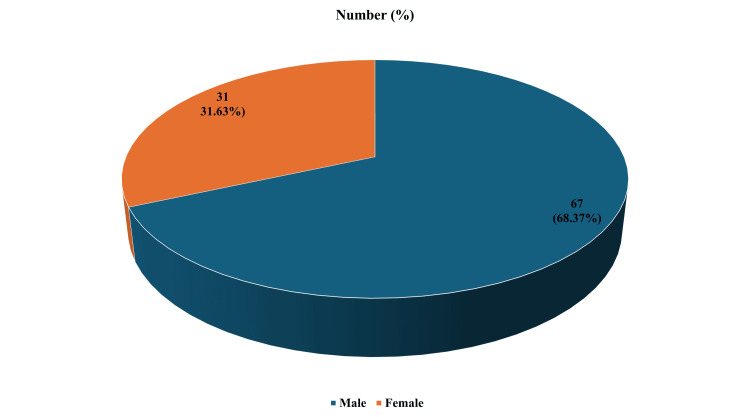
Gender of patients included in the study. The image is created by the author. Number of admitted male and female patients over the two years of study duration (April 2020 and July 2025), along with the percentage proportion of total patients for each gender in brackets.

A total of 98 patients met the inclusion criteria, and 234 ophthalmological signs and symptoms were studied. The mean age of the study population was 47 ± 21.5 years, comprising 67 (68.37%) males and 31 (31.63%) females, as shown in Figure 1.

The most common otorhinolaryngological condition presenting with ophthalmological manifestations was Chronic Rhinosinusitis (CRS), observed in 12 cases (12.24%). This was followed by orbital cellulitis (11 cases, 11.22%), chronic otitis media (COM) with complications (10 cases, 10.20%), trauma (8 cases, 8.16%), and acute otitis media (AOM)/mastoiditis (8 cases, 8.16%). The full list of conditions with their respective numbers and percentages is presented in Table 1.

**Table 1:** Number of patients with different otorhinolaryngologic head and neck diagnoses. Total number of cases (N): 98 (100%)

Diagnosis	Number of Patients	Percentage (%)
Chronic Rhinosinusitis	12	12.24
Orbital Cellulitis (non-specific causes)	11	11.22
Chronic Otitis Media with Complications	10	10.20
Acute Invasive Fungal Rhinosinusitis	9	9.18
Trauma	8	8.16
Acute Otitis Media/Mastoiditis	8	8.16
Acute Bacterial Rhinosinusitis	7	7.14
Squamous Cell Carcinoma Maxillary Sinus	7	7.14
Parotid Malignancies	7	7.14
Allergic Fungal Rhinosinusitis	5	5.10
Adenocarcinoma Ethmoid Sinus	4	4.08
Parapharyngeal Space Mass	3	3.06
Other Sino-nasal Malignancies	2	2.04
Temporal Bone Malignancy	2	2.04
Thyroid disease	2	2.04
Rhabdomyosarcoma Sinus and Orbit	1	1.02

**Figure 2:**
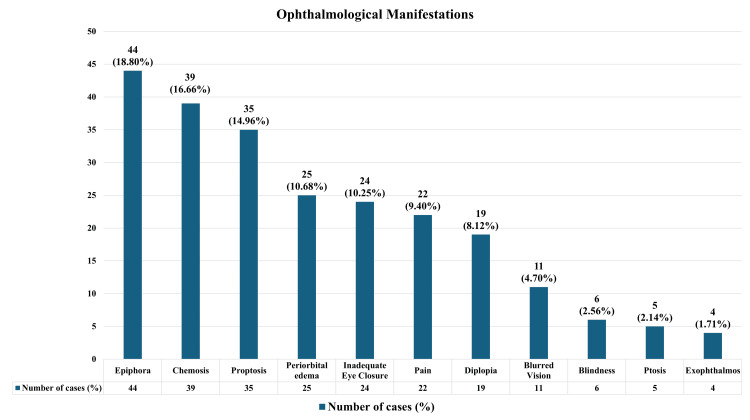
Ophthalmologic manifestation of different signs and symptoms. The image is created by the author. y-axis (number of cases): The number of cases corresponding to the specific sign and symptom. x-axis (Ophthalmologic manifestations): The different signs and symptoms associated with ophthalmologic manifestations.

Various ophthalmological signs and symptoms were observed across different otorhinolaryngological conditions, with some diseases exhibiting more than one manifestation. Considering all cases, the most common ophthalmological manifestation was epiphora, seen in 44 cases (18.80%), followed by chemosis in 39 cases (16.66%). Other frequent manifestations included periorbital edema (25 cases, 10.68%), inadequate eye closure/Bell’s phenomenon (24 cases, 10.25%), pain (22 cases, 9.40%), and diplopia (19 cases, 8.12%). A detailed summary of all ophthalmological manifestations, along with their frequencies and percentages, is illustrated in Figure 2.

## Discussion

The patients commonly present with multiple ophthalmological complaints associated with their otorhinolaryngological disorders. The most frequent manifestations were epiphora, observed in 44 cases (18.80%), followed by chemosis in 39 cases (16.66%).

The mean age of the patients was 47 ± 21.5 years, with a male predominance of 67 (68.37%) compared to 31 (31.63%) females, consistent with findings reported by Manjumol S et al. [7], Chavan SS et al. [8], and Ghosh D et al. [1]. The most common ophthalmological manifestations identified, epiphora, chemosis, and periorbital edema, were also frequently reported in other studies. For example, Manjumol S et al. [7] documented periorbital edema in 51.6% and epiphora in 48.3% of cases. Novshaba et al. [9] reported proptosis as the most common symptom, with a prevalence of 31%. Ghosh D et al. [1] found lagophthalmos in 30.64% and proptosis in 20.96% of patients, comparable to our findings. Qazi ZU et al. [3] observed diplopia in 23%, vision loss in 16%, and ptosis in 15%, which aligns closely with our results. Kumar A et al. [10] reported proptosis in 44.73% of cases, while Sinha V et al. [11] concluded that proptosis is the most frequent clinical presentation in neoplastic lesions of the nose and paranasal sinuses.

Chronic rhinosinusitis (CRS) was the most common otorhinolaryngological condition associated with ophthalmological manifestations in our cohort, affecting 12 patients (12.24%), followed by orbital cellulitis (11 cases, 11.22%), chronic otitis media (COM) with complications (10 cases, 10.20%), trauma (8 cases, 8.16%), and acute otitis media (AOM)/Mastoiditis (8 cases, 8.16%). Though the prevalence rates vary, these findings are consistent with prior studies. Manjumol S et al. [7] reported CRS in 34.9% and orbital cellulitis in 47% of their cases. Sabharwal KK et al. [12] noted incidences of maxillary sinus carcinoma and other tumors at 6% and 4%, respectively, compared to 7% and 6% in our study; Ghosh D et al. [1] reported 23% and 7%, respectively. While Zeyad et al. [13] described orbital cellulitis as rare, it was observed in 11.22% of our cases, likely reflecting geographical differences. Adenocarcinoma of the ethmoid causing orbital involvement was seen in 7.14% of cases in Ghosh D et al.’s study [1], compared to 4.08% in ours.

Further, Manjumol S et al. [7] reported allergic fungal rhinosinusitis (AFRS) in 15% and sinonasal carcinoma in 7.2% of patients, aligning with our results. Qazi ZU et al. [3] found squamous cell carcinoma (SCC) in 4%, adenocarcinoma in 3%, acute rhinosinusitis in 2%, and rhabdomyosarcoma in 1%, consistent with our findings. Venugopal et al. [14] described fungal sinusitis in 16% of cases, matching our combined rate for all fungal rhinosinusitis. Elango S et al. [15] reported the incidence of orbital complications from sinusitis ranging from 0.5% to 3.9%, which differs from the rates observed in our study.

This study highlights the spectrum and trends of ophthalmologic manifestations associated with otorhinolaryngology-head and neck diseases, enhancing understanding of the ophthalmo-otorhinolaryngological relationship. These insights can improve diagnosis, clinical decision-making, and patient management. The strengths of this study include its five-year duration and inclusion of formally diagnosed inpatients. However, limitations include a relatively small sample size, single-center design, retrospective methodology, and the rarity of some conditions studied, which may affect the generalizability of our findings.

## Conclusions

The most common ophthalmologic manifestations of otorhinolaryngology-head and neck diseases are epiphora and chemosis. Among the ENT conditions, chronic rhinosinusitis and non-specific orbital cellulitis are the most frequently associated with ophthalmological signs and symptoms. This study highlights the importance of a high index of suspicion among ophthalmologists to consider causes beyond primary eye diseases when evaluating ophthalmological presentations. Similarly, otorhinolaryngologists should remain vigilant for non-ENT symptoms that arise secondary to diseases of the ear, nose, and throat. Early diagnosis, timely referral, and effective collaboration between specialists are crucial in preserving vision and saving lives. Therefore, close interdisciplinary cooperation between ophthalmology and otorhinolaryngology departments is essential for the successful management of these patients.
